# Intentions and Attempts to Quit JUUL E-Cigarette Use: The Role of Perceived Harm and Addiction

**DOI:** 10.5888/pcd19.210255

**Published:** 2022-02-03

**Authors:** Andréa L. Hobkirk, Brianna Hoglen, Tianhong Sheng, Ava Kristich, Jessica M. Yingst, Kenneth R. Houser, Nicolle M. Krebs, Sophia I. Allen, Candace R. Bordner, Craig Livelsberger, Jonathan Foulds

**Affiliations:** 1 Department of Psychiatry and Behavioral Health, Pennsylvania State University College of Medicine, Hershey, Pennsylvania; 2 Department of Public Health Sciences, Pennsylvania State University College of Medicine, Hershey, Pennsylvania; 3 Center for Research on Tobacco and Health, Pennsylvania State University College of Medicine, Hershey, Pennsylvania; 4 Department of Statistics, Pennsylvania State University, State College, Pennsylvania

## Abstract

**Introduction:**

Research on electronic cigarette (e-cigarette) quit intentions and attempts is limited despite the potential health benefits of quitting, especially for long-term users. The current study aimed to investigate perceptions of harm and addictiveness and tobacco use characteristics associated with quit variables among users of a popular e-cigarette brand, JUUL.

**Methods:**

We surveyed 301 US adult JUUL users on their tobacco use characteristics, perceptions of JUUL harm and addictiveness, and quit variables at 3 time points, from July 2019 to April 2020. We used logistic regression models to assess demographic characteristics, smoking characteristics, and perceptions of JUUL harm and addictiveness as correlates of e-cigarette quit intentions, attempts, importance, and confidence.

**Results:**

Twenty-three percent of the sample had intentions to quit using JUUL within the year, and 22.6% reported making a lifetime quit attempt. The average rating of quit importance was 4.1 and quit confidence was 5.8 on a Likert scale of 1 to 10. More than 90% of the sample indicated that JUUL was at least moderately addictive, whereas less than one-quarter indicated that JUUL was as harmful or more harmful than smoking. Higher levels of perceived JUUL addictiveness were associated with more quit intentions, attempts, and importance. Higher levels of perceived JUUL harm compared with smoking were associated with more quit importance.

**Conclusion:**

Our findings suggest that a small proportion of adult JUUL users are interested in quitting. Self-reported perceptions of JUUL’s addiction potential may be related to more quit attempts. Findings highlight the need for evidence-based information on e-cigarette addictiveness and effective strategies for cessation.

**Summary**

**What is already known on this topic?**

Interest in e-cigarette use cessation is growing among regular users, but data on adult e-cigarette users are limited. Qualitative research among young e-cigarette users shows that perceived harm and addictiveness are linked to quit intentions.

**What is added by this report?**

The current study provides the first quantitative assessment of e-cigarette quit intentions and attempts, and factors associated with quit variables, including perceived e-cigarette harm and addictiveness, among adult users.

**What are the implications for public health practice?**

Although levels of quit intentions and attempts are low among adult e-cigarette users, those with self-reported symptoms of dependence and perceptions that e-cigarettes are more addictive than smoking may benefit from e-cigarette cessation treatments.

## Introduction

An estimated 8.1 million adults currently use electronic cigarettes (e-cigarettes) in the US (1). Unlike cigarettes, which use combustion to generate tobacco smoke, e-cigarettes aerosolize a nicotine-containing e-liquid for inhalation (2), resulting in lower intake of carcinogens and toxins for long-term e-cigarette users compared with smokers (3,4). Although replacing cigarettes with e-cigarettes likely reduces health risks in the short term for current smokers, e-liquids can contain high levels of nicotine and toxic compounds that may contribute to addiction and respiratory damage (5,6).

Adults’ perceptions that e-cigarettes are harmful and addictive have increased over the past decade along with increased messaging about harms (7–9). For example, the Centers for Disease Control and Prevention website states that e-cigarettes contain toxic and carcinogenic chemicals, cause long-lasting changes in the brain, and are not a method for smoking cessation approved by the US Food and Drug Administration (FDA) (eg, cdc.gov/ecigarettes; drugabuse.gov/tobacconicotine-vaping). Data from several national surveys collected in the past decade revealed that the percentage of US adults who believe e-cigarettes are less harmful than cigarettes decreased, while the percentage who believe that e-cigarettes are addictive doubled (7–10).

Motivation for e-cigarette use cessation is rising in parallel with attention to the potential harms of e-cigarettes and the growing numbers of long-term e-cigarette users. Online and national surveys of adult e-cigarette users found that approximately 30% to 60% expressed some interest in quitting and 10% to 64% had made a previous e-cigarette quit attempt (11–13). However, research is limited on the factors associated with e-cigarette quit intentions and attempts, including the influence of harm or addiction perceptions.

Given the changing landscape of e-cigarette harm messaging and regulations, and the media attention given to e-cigarettes, the primary objective of the current study was to investigate e-cigarette quit intentions and attempts by identifying associated factors and focusing on adult JUUL users. Of all e-cigarette brands, JUUL has received the most negative attention because of its popularity among adolescents and young adults and its relatively high levels of nicotine (14). Therefore, we hypothesized that perceptions of JUUL’s addictive potential and harm relative to cigarettes would be associated with quit intentions and attempts.

## Methods

In this cross-sectional survey, we used Amazon Mechanical Turk (MTurk) to recruit adults aged 18 or older who indicated having used JUUL in the past 30 days. MTurk is an online labor market where individuals with a registered account, called workers, can complete online jobs, such as computer tasks and surveys, for compensation. Workers can choose from a list of jobs available to them based on their demographic characteristics and geographic location. Substance users and young adults tend to be overrepresented among MTurk workers compared with the general population, making MTurk an ideal platform for research on e-cigarette use behavior (15). To quantify workers’ performance, MTurk assigns each worker a job approval rating based on their number of successfully completed jobs. To improve the reliability and validity of the current survey, we implemented a requirement that only US workers with job approval ratings of at least 98% were eligible to participate. We conducted the cross-sectional survey at 3 time points, July 2019, January 2020, and April 2020, with different samples at each point. All participants provided informed consent electronically before completing study procedures and were compensated $2 for participation. This payment is consistent with MTurk standards (16). All procedures were approved by the Penn State College of Medicine Institutional Review Board.

### Measures

The 30-minute survey was developed by the study team to assess JUUL use behavior. The survey assessed demographic information with the items, “How old are you?” and “Are you male or female?”; JUUL use characteristics, including “How long have you been using an electronic cigarette?” and “How long have you been using a JUUL electronic cigarette?” with options to respond in the number of days, months, or years; “How many days in the last 30 have you used your JUUL electronic cigarette?”; “In the past 30 days, what nicotine concentration JUUL pods did you purchase/use?” with response options “5%,” “3%,” or “both”; “Where did you purchase your JUUL electronic cigarette?” with response options “gas station,” “vape shop,” “tobacco store,” “online,” or “I did not purchase”; and “When was the last time you smoked a cigarette?” with options to respond in the number or days, months, or years. Former smokers were asked, “Did you quit smoking before or after you started using JUUL?” with response options “I quit cigarette smoking before I started using JUUL or any other electronic cigarette,” “I quit cigarette smoking after I started using an e-cig other than JUUL,” and “I quit cigarette smoking after I started using JUUL.” We measured e-cigarette dependence with the Penn State Electronic Cigarette Dependence Index (PSECDI); total scores for this index range from 0 to 20, with higher scores indicating greater dependence. Levels of dependence were categorized according to the PSECDI as not dependent (0–3), low dependence (4–8), medium dependence (9–12), and high dependence (≥13) (17). The PSECDI has normative data from more than 3,600 e-cigarette users and shows construct validity in that scores are related to the nicotine concentration of liquids used and show convergent validity with the E-cigarette dependence scale among exclusive e-cigarette users (*r* = 0.71) (18). We used the following PSECDI item as an independent correlate in our analyses: “How many times per day do you use your JUUL electronic cigarette? (assume that one time consists of around 15 puffs or lasts around 10 minutes).”

Items assessing the quit variables included 1) “Are you planning to continue using your JUUL electronic cigarette for at least the next year, or quit within that time frame?” with response options “I’ll quit using JUUL within a year,” “I plan to continue using the JUUL,” or “Don’t know”; 2) “How important is it for you to quit electronic cigarette use now?” on a 10-point Likert scale ranging from “not at all” to “very”; 3) “How confident are you that you could quit electronic cigarette use now?” on a 10-point Likert scale ranging from 1 (“not at all”) to 10 (“very”); and 4) “Have you ever tried to quit using your JUUL electronic cigarette?” with response options no or yes. Those who made a quit attempt were asked the duration of their quit attempt and if they used any of the following methods during the attempt: “nicotine replacement therapy,” “cold turkey,” “used another tobacco product,” or “FDA-approved medication like Zyban or Chantix.” Participants responded to 2 questions about their perceptions of JUUL use: 1) “Overall, how addictive do you believe using a JUUL is?” with response options “not at all addictive,” “moderately addictive,” and “very addictive” and 2) “Compared to smoking, would you say that JUUL use is . . . ” with response options “much less harmful,” “less harmful,” “just as harmful,” or “much more harmful.”

The survey also inquired about education and employment, e-liquid and pod purchases and refilling behaviors, physical and health effects of JUUL use, motivations for JUUL use, and use of other tobacco products. Information from respondents on the use of flavored JUUL pods in the context of flavor regulations is available elsewhere (19).

### Data cleaning

To exclude respondents who were not authentic JUUL users, we required all participants to submit a nonstock photograph of their JUUL device. We excluded from analysis respondents who submitted a stock or non-JUUL photo and illogical or random responses (n = 98). In addition, we removed from analysis 22 duplicate responses across the 3 time points. In total, we excluded 120 respondents from analysis, which resulted in a final sample of 301 unique respondents.

### Data analysis

We used SPSS version 26 (IBM Corp) to calculate means and frequencies and to conduct 1-way analyses of variance and χ^2^ tests for all demographic, perception, and quit variables by smoking status. To prepare the data for regression analysis, we normalized positively skewed variables by applying a square root transformation to the number of months of e-cigarette and JUUL use and a natural log transformation to the number of JUUL use times per day. For smoking status, current smokers were those that indicated using a cigarette less than 1 month ago. Former smokers indicated last using a cigarette 1 month ago or longer. Never smokers reported never using a cigarette. We categorized scores on self-reported quit importance and confidence into low (1–3), medium (4–7), and high (8–10) to simplify analysis. We conducted binomial and multinomial logistic regression models to examine 1) demographic and smoking characteristics as correlates of harm and addiction perceptions and 2) demographic and smoking characteristics and harm and addiction perceptions as correlates of quit variables (ie, intentions, attempts, importance, and confidence). The correlates assessed were sex, age, survey collection time point, place of device purchase, smoking status (ie, current, former, never), PSECDI level, number of use times per day, days of use in the past 30, months of e-cigarette use, months of JUUL use, perceived harm, and perceived addictiveness. We treated all correlates as fixed effects in the models. We first fit each model by using all correlates and then selected models based on the Akaike information criterion (AIC). The AIC considers the risk of overfitting and underfitting to converge on a final model with good fit for the data and simplicity. We calculated total fit metrics (AIC and residual deviance) for each model. We calculated analysis of deviance tables to display the significance of each correlate in the final models with χ^2^ tests. We calculated odds ratios and significance values of each model parameter estimate to assist with interpretation; however, some subgroup sample sizes were too small to produce reliable parameter estimates, and therefore should be interpreted with caution. We conducted regression analyses by using R statistical software version 3.4.2 (R Foundation for Statistical Computing).

## Results

The final sample included 301 current JUUL users; 36.2% (n = 109) were women, and the mean age was 31.9 (SD, 8.5) (Table 1). The sample included 55 (18.3%) current smokers, 165 (54.8%) former smokers, and 81 (26.9%) never smokers. Former smokers last smoked a cigarette 24.6 (SD, 30.7) months ago on average; 35% (58 of 165) of former smokers reported that they quit using cigarettes after starting to use a JUUL. Half of the sample (51%, n = 154) reported using their JUUL daily with an average of 11.8 (SD, 37.9) times per day. Self-reported JUUL use ranged from 1 to 500 times per day among the total sample. Four participants reported using their JUUL 100 or more times per day.

**Table 1:** Demographic Characteristics, JUUL Use, JUUL Perceptions, and Quit Variables Across Smoking Status for a Sample of US Adult JUUL Users, 2019–2020^a^

Characteristic	Never smoker (n = 81)	Current smoker (n = 55)	Former smoker (n = 165)	Total sample (N = 301)	*P* value^b^
**Demographic**					
Female sex, n (%)	35 (43.2)	13 (23.6)	61 (37.0)	109 (36.2)	.06
Age, mean (SD), y	28.8 (8.0)	35.2 (9.5)	32.3 (7.9)	31.9 (8.5)	<.001
**JUUL use**					
No. of months have used e-cigarettes, mean (SD)	20.9 (21.8)	28.7 (22.4)	29.8 (24.6)	27.2 (23.7)	.02
No. of months have used JUUL, mean (SD)	13.5 (13.0)	13.4 (12.5)	14.0 (10.6)	13.8 (11.6)	.93
JUUL use days in past 30 days, mean (SD)	21.1 (8.6)	22.8 (8.7)	24.5 (8.3)	23.3 (8.6)	.01
JUUL use times per day, mean (SD)	10.3 (17.4)	16.2 (53.3)	11.2 (39.1)	11.8 (37.9)	.63
Where purchase JUUL, n (%)					
Gas station	22 (27.2)	22 (40.0)	63 (38.2)	107 (35.5)	.06
Vape shop	28 (34.6)	9 (16.4)	52 (31.5)	89 (29.6)	
Tobacco store	7 (8.6)	9 (16.4)	14 (8.5)	30 (10.0)	
Online	23 (28.4)	11 (20.0)	30 (18.2)	64 (21.3)	
Did not purchase	1 (1.2)	4 (7.3)	6 (3.6)	11 (3.7)	
Nicotine concentration, n (%)					
5% concentration	32 (42.7)	33 (63.5)	81 (51.9)	146 (51.6)	.07
3% concentration	31 (41.3)	11 (21.2)	42 (26.9)	84 (29.7)	
Both	12 (16.0)	8 (15.4)	33 (21.2)	53 (18.7)	
PSECDI total score, mean (SD)^c^	8.0 (4.5)	9.1 (4.4)	8.4 (4.0)	8.4 (4.2)	.31
PSECDI dependence level, n (%)					
Not dependent	17 (21.0)	8 (14.5)	20 (12.1)	45 (15.0)	.43
Low dependence	27 (33.3)	15 (27.3)	63 (38.2)	105 (34.9)	
Medium dependence	25 (30.9)	19 (34.5)	53 (32.1)	97 (32.2)	
High dependence	12 (14.8)	13 (23.6)	29 (17.6)	54 (17.9)	
**JUUL perceptions**					
How addictive, n (%)					
Not at all addictive	14 (17.3)	4 (7.3)	6 (3.6)	24 (8.0)	.002
Moderately addictive	54 (66.7)	37 (67.3)	111 (67.3)	202 (67.1)	
Very addictive	13 (16.0)	14 (25.5)	48 (29.1)	75 (24.9)	
How harmful compared with smoking, n (%)					
Much less harmful	30 (37.0)	8 (14.5)	43 (26.1)	81 (26.9)	.04
Less harmful	29 (35.8)	31 (56.4)	88 (53.3)	148 (49.2)	
Just as harmful	18 (22.2)	13 (23.6)	31 (18.8)	62 (20.6)	
Much more harmful	4 (4.9)	3 (5.5)	3 (1.8)	10 (3.3)	
**Quit variables**					
Quit attempts, n (%)					
No	52 (64.2)	44 (80.0)	137 (83.0)	233 (77.4)	.004
Yes	29 (35.8)	11 (20.0)	28 (17.0)	68 (22.6)	
**Quit intentions, n (%)**					
I’ll quit JUUL within a year	17 (21.0)	10 (18.2)	41 (24.8)	68 (22.6)	.55
I will continue to use/don’t know	64 (79.0)	45 (81.8)	124 (75.2)	233 (77.4)	
Quit importance, mean (SD)^d^	4.1 (3.0)	4.1 (2.8)	4.2 (2.6)	4.1 (2.8)	.98
Quit confidence, mean (SD)^d^	6.4 (2.7)	5.6 (2.8)	5.6 (2.7)	5.8 (2.7)	.07

Abbreviation: PSECDI, Penn State Electronic Cigarette Dependence Index.

a Data source: A 30-minute survey was developed by the study team and administered at 3 time points: July 2019, January 2020, and April 2020.

b *P* values determined from variable comparisons across smoking status using 1-way analyses of variance and χ^2^ tests.

c Scores range from 0 to 20, where higher scores indicate greater dependence. Levels of dependence were categorized as not dependent (0–3), low dependence (4–8), medium dependence (9–12), and high dependence (≥13) (17).

d A 10-point Likert scale ranging from “not at all” to “very.” Scores categorized into low (1–3), medium (4–7), and high (8–10) to simplify analysis.

### Total sample quit variables and harm perceptions

Less than one-quarter (n = 68; 22.6%) indicated an intention to quit using JUUL within the year. The sample rated a mean of 4.1 (SD, 2.8) of 10 for the importance of quitting and a mean of 5.8 (SD, 2.7) of 10 for confidence in their ability to quit. Of the 68 (22.6%) who made a lifetime JUUL quit attempt, the average attempt lasted 2.4 months (SD, 3.4) and almost half (n = 31; 45.6%) reported an attempt that lasted less than a month. Of those who reported a quit attempt, most (n = 46; 67.6%) reported quitting JUUL “cold turkey” with the remainder reporting use of nicotine replacement therapy (n = 11; 16.2%), another tobacco product (n = 7; 10.3%), or an FDA-approved medication (n = 4; 5.9%). 

Two-thirds of the sample indicated that JUUL was moderately addictive (n = 202; 67.1%) and one-quarter indicated that JUUL was very addictive (n = 75; 24.9%); 76.1% (n = 229) of the sample believed that JUUL was less or much less harmful than smoking, and only 10 (3.3%) participants believed that JUUL was much more harmful than smoking (Table 1).

**Figure 1:**
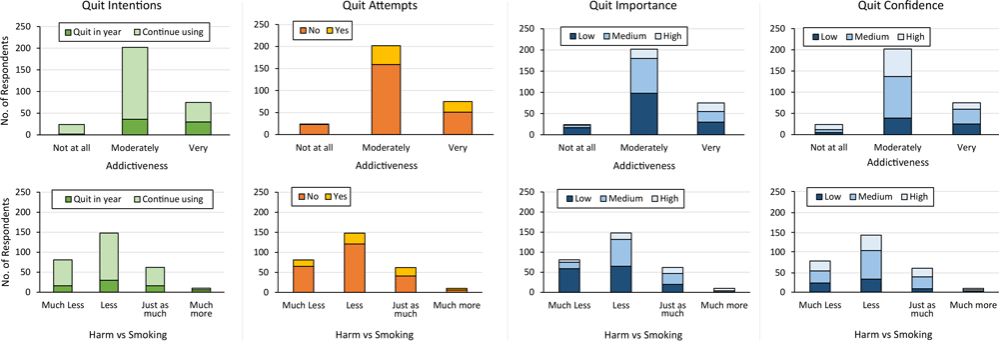
Frequency of quit variables by harm and addiction perceptions among a sample of US adult JUUL users (N = 301), 2019–2020.

**Figure 2:**
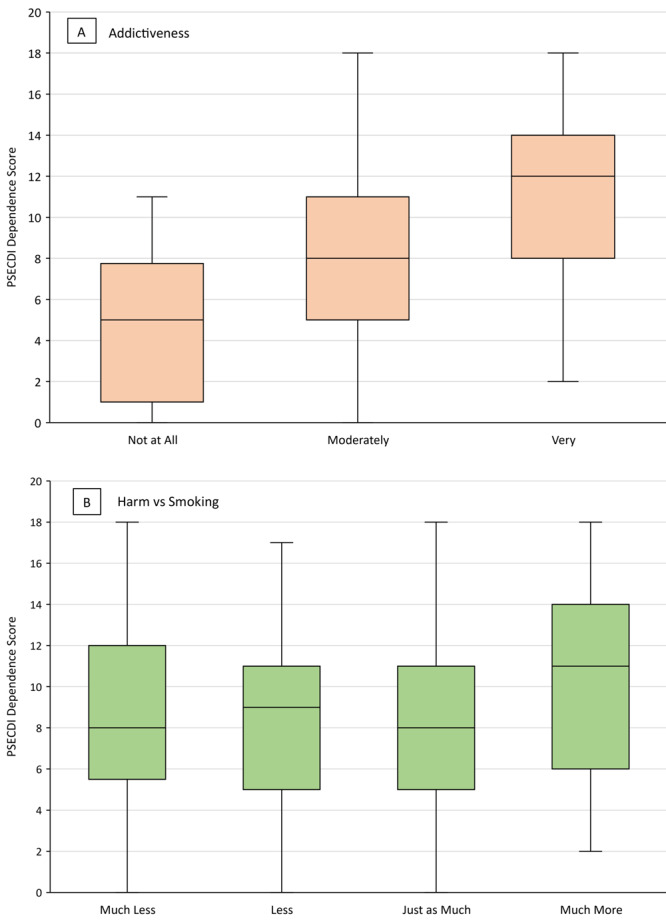
Mean PSECDI dependence score by level of A) perceived addictiveness of JUUL and B) perceived harm (compared with smoking) of JUUL among a sample of US adult JUUL users (N = 301), 2019–2020. Boxes indicate the 25th percentile, median, and 75th percentile. Whiskers show the minimum and maximum ranges. Abbreviation: PSECDI, Penn State Electronic Cigarette Dependence Index.

Of the 75 JUUL users who believed use was very addictive, 45 (60.0%) indicated they would continue using JUUL (Figure 1). Among JUUL users who believed use was very addictive, the median PSECDI score was 12.0 (range, 2–18), and among users who believed use was not at all addictive, the median PSECDI score was 5 (range, 0–11) (Figure 2). Among JUUL users who believed use was much more harmful than smoking, the median score was 11 (range, 2–18), and among users who believed use was much less harmful than smoking, the median score was 8 (range, 0–18).

**Table:** 

Quit Outcome	Addictiveness, No. (%)			Harm, Compared With Smoking, No. (%)			
	Not at all	Moderately	Very	Much Less	Less	Just as Much	Much More
**Intention**							
Quit in year	2 (8.3)	36 (17.8)	30 (40.0)	16 (19.8)	30 (20.3)	16 (25.8)	6 (60.0)
Continue using	22 (91.7)	166 (82.2)	45 (60.0)	65 (80.2)	118 (79.7)	46 (74.2)	4 (40.0)
**Attempt**							
No	23 (95.8)	159 (78.7)	51 (68.0)	65 (80.2)	121 (81.8)	41 (66.1)	6 (60.0)
Yes	1 (4.2)	43 (21.3)	24 (32.0)	16 (19.8)	27 (18.2)	21 (33.9)	4 (40.0)
**Importance**							
Low	17 (70.8)	98 (48.5)	30 (40.0)	59 (72.8)	65 (43.9)	20 (32.3)	1 (10.0)
Medium	6 (25.0)	82 (40.6)	25 (33.3)	16 (19.8)	67 (45.3)	27 (43.5)	3 (30.0)
High	1 (4.2)	22 (10.9)	20 (26.7)	6 (7.4)	16 (10.8)	15 (24.2)	6 (60.0)
**Confidence**							
Low	5 (20.8)	39 (19.3)	25 (33.3)	24 (29.6)	34 (23.0)	9 (14.5)	2 (20.0)
Medium	7 (29.2)	98 (48.5)	35 (46.7)	31 (38.3)	74 (50.0)	31 (50.0)	4 (40.0)
High	12 (50.0)	65 (32.2)	15 (20.0)	26 (32.1)	40 (27.0)	22 (35.5)	4 (40.0)

**Table:** 

PSECDI Dependence Score	Perceived Addictiveness			Perceived Harm, Compared With Smoking			
	Not at all	Moderately	Very	Much Less	Less	Just as Much	Much More
Median	5	8	12	8	9	8	11
75th percentile	7.8	11	14	12	11	11	14
25th percentile	1	5	8	5.5	5	5	6
Minimum	0	0	2	0	0	0	2
Maximum	11	18	18	18	17	18	18

### Regression models of JUUL perceptions and quit variables

In the final logistic regression models, factors associated with higher perceived JUUL addictiveness included higher PSECDI score, being female (vs male), and being a current or former smoker (vs never smoker) (Table 2). Older age was associated with a higher likelihood of endorsing JUUL as moderately (vs not at all) addictive but a lower likelihood of endorsing JUUL as very (vs not at all) addictive. Factors associated with higher perceived JUUL harm compared with smoking included fewer lifetime months of e-cigarette use, being a current or former smoker (vs never smoker), and being female (vs male) (Table 3). Factors associated with intentions to quit JUUL use in the next year included higher perceived addictiveness and fewer times per day of JUUL use (Table 4). Factors associated with a lifetime quit attempt included higher perceived addictiveness, higher PSECDI score, being a never smoker (vs current or former smoker), and fewer days of JUUL use in the past 30 days (Table 5). Factors associated with higher quit importance included higher perceived addictiveness and harm, higher PSECDI score, being in the second or third collection time point (vs first time point), fewer days of JUUL use in the past 30 days, and fewer lifetime months of e-cigarette use (Table 6). Place of JUUL purchase was a relevant correlate of perceived quit importance, but the direction was difficult to interpret because of sample sizes for the purchase categories. Lower dependence level was the only significant correlate of higher quit confidence.

**Table 2:** Results of Logistic Regression Models of JUUL Addictiveness Perception Among US Adult JUUL Users (N = 301), 2019–2020^a^

Variable	χ^2^ *P* value	Odds ratio (95% CI)	
		Moderately vs not at all	Very vs not at all
Age	.001	1.05 (0.10–1.12)	0.98 (0.09–1.05)
Sex			
Male	.004	0.95 (0.03–1.12)	0.34 (0.01–1.06)
Female		1 [Reference]	1 [Reference]
Smoking status			
Current	.002	1.42 (0.04–5.49)	4.02 (0.08–20.19)
Former		4.13 (1.43–11.94)^b^	11.66 (3.25–41.88)^c^
Never		1 [Reference]	1 [Reference]
PSECDI dependence level^d^			
Low	<.001	1.69 (0.06–4.60)	5.46 (0.94–31.55)
Medium		6.31 (1.57–25.39)^b^	38.33 (5.33–275.41)^c^
High		2,356,778 (1,077,123–5,156,700)^c^^,^ e	44,203,672 (2,020,259–9,671,854)^c^^,^ e
Not dependent		1 [Reference]	1 [Reference]

Abbreviation: PSECDI, Penn State Electronic Cigarette Dependence Index.

a A 30-minute survey was developed by the study team and administered at 3 time points: July 2019, January 2020, and April 2020. The question on addictiveness was, “Overall, how addictive do you believe using a JUUL is?” with response options “not at all addictive,” “moderately addictive,” and “very addictive” Estimates of each variable level were determined by using binomial and multinomial logistic regression models. Akaike information criterion = 432.9; residual deviance = 400.9.

b *P* < .01; determined by χ^2^ test.

c *P* < .001; determined by χ^2^ test.

d Scores range from 0 to 20, where higher scores indicate greater dependence. Levels of dependence were categorized as not dependent (0–3), low dependence (4–8), medium dependence (9–12), and high dependence (≥13) (17).

e The odds ratios for high dependence are large because we modeled the log-odds as a linear function of each variable and, therefore, applied an exponential function to the odds ratio calculations. These estimates are reliable and not biased by outliers.

**Table 3:** Results of Logistic Regression Models of Perceived Harm of JUUL, Compared With Smoking, Among US Adult JUUL Users (N = 301), 2019–2020^a^

Variable	χ^2^ *P* value	Odds ratio (95% CI)		
		Less vs much less harmful	Just as much vs much less harmful	Much more vs much less harmful
Sex				
Male	.01	1.20 (0.66–2.17)	0.72 (0.35–1.47)	0.26 (0.06–1.09)
Female		1 [Reference]	1 [Reference]	1 [Reference]
Smoking status				
Current	.02	4.56 (1.75–11.88)^b^	3.81 (1.26–11.52)^c^	3.78 (0.64–22.45)
Former		2.55 (1.32–4.94)^b^	1.71 (0.77–3.78)	0.65 (0.12–3.46)
Never		1 [Reference]	1 [Reference]	1 [Reference]
Days of JUUL use in past 30 days	.09	0.98 (0.94–1.01)	0.96 (0.92–1.00)	0.92 (0.85–0.99)^c^
Months of e-cigarette use, square root	.046	0.90 (0.79–1.02)	0.83 (0.70–0.97)^c^	1.12 (0.83–1.51)

a A 30-minute survey was developed by the study team and administered at 3 time points: July 2019, January 2020, and April 2020. The question on harm perception was, “Compared to smoking, would you say that JUUL use is . . . ” with response options “much less harmful,” “less harmful,” “just as harmful,” or “much more harmful.” Estimates of each variable level were determined by using binomial and multinomial logistic regression models. Akaike information criterion = 688.6; residual deviance = 652.6.

b *P* < .01; determined by χ^2^ test.

c *P* < .001; determined by χ^2^ test.

**Table 4:** Results of Logistic Regression Models of Quit Intentions Among US Adult JUUL Users (N = 301), 2019–2020^a^

Variable	χ^2^ *P* value	I’ll quit vs I’ll continue
Perceived addictiveness of JUUL		
Moderately addictive	<.001	3.36 (0.88–22.35)
Very addictive		12.46 (2.92–88.24)^b^
Not at all		1 [Reference]
Perceived harm of JUUL, compared with smoking		
Less harmful	.14	0.68 (0.33–1.47)
Just as much harm		0.86 (0.36–2.03)
Much more harm		3.37 (0.77–15.91)
Much less harm		1 [Reference]
JUUL use times per day, log	.03	0.70 (0.49–0.97)^c^
Months of JUUL use	.51	1.08 (0.85–1.38)
Months of e-cigarette use	.05	0.85 (0.72–1.00)
Survey time point		
Time point 2	.06	2.26 (1.01–5.48)
Time point 3		2.75 (1.16–7.00)^c^
Time point 1		1 [Reference]

a A 30-minute survey was developed by the study team and administered at 3 time points: July 2019, January 2020, and April 2020. The question on quit intentions was, “Are you planning to continue using your JUUL electronic cigarette for at least the next year, or quit within that time frame?” with response options “I’ll quit using JUUL within a year,” “I plan to continue using the JUUL,” or “Don’t know.” Estimates of each variable level were determined by using binomial and multinomial logistic regression models. Akaike information criterion = 306.5; residual deviance = 284.5.

b *P* < .01.

c *P* < .05.

**Table 5:** Results of Logistic Regression Models of Quit Attempts Among US Adult JUUL Users (N = 301), 2019–2020^a^

Variable	χ^2^ *P* value	Yes vs no
Perceived addictiveness of JUUL		
Moderately addictive	.03	5.64 (0.99–107.16)
Very addictive		10.56 (1.62–212.12)^b^
Not at all		1 [Reference]
Perceived harm of JUUL, compared with smoking		
Less harmful	.10	0.68 (0.30–1.52)
Just as much harm		1.83 (0.76–4.49)
Much more harm		0.97 (0.17–5.26)
Much less harm		1 [Reference]
Smoking status		
Current	.007	0.35 (0.14–0.87)^b^
Former		0.32 (0.15–0.66)^c^
Never		1 [Reference]
PSECDI dependence level^d^		
Low	<.001	2.96 (0.81–14.52)
Medium		15.16 (4.26–75.04)^e^
High		8.85 (2.18–47.39)^c^
Not dependent		1 [Reference]
Days of JUUL use in past 30 days	.002	0.94 (0.90–0.98)^c^

Abbreviation: PSECDI, Penn State Electronic Cigarette Dependence Index.

a A 30-minute survey was developed by the study team and administered at 3 time points: July 2019, January 2020, and April 2020. The question on quit attempt was, “Have you ever tried to quit using your JUUL electronic cigarette?” with response options no or yes. Estimates of each variable level were determined by using binomial and multinomial logistic regression models. Akaike information criterion = 280.9; residual deviance = 256.9.

b *P* < .05.

c *P* < .01.

d Scores range from 0 to 20, where higher scores indicate greater dependence. Levels of dependence were categorized as not dependent (0–3), low dependence (4–8), medium dependence (9–12), and high dependence (≥13) (17).

e *P* < .001.

**Table 6:** Results of Logistic Regression Models of Quit Importance and Quit Confidence Among US Adult JUUL Users (N = 301), 2019–2020^a^

Variable	χ^2^ *P* value	Moderate vs low	High vs low
**Quit importance^b^**			
Survey time point			
Time point 2	.04	2.21 (1.07–4.56)^c^	1.16 (0.41–3.33)
Time point 3		3.40 (1.56–7.42)^d^	1.29 (0.42–3.99)
Time point 1		1 [Reference]	1 [Reference]
Age	.09	0.99 (0.96–1.02)	1.04 (0.99–1.09)
Place of JUUL purchase			
Vape shop	.03	1.03 (0.50–2.10)	1.20 (0.42–3.38)
Tobacco store		0.75 (0.28–2.02)	0.62 (0.15–2.48)
Online		0.54 (0.25–1.18)	0.19 (0.42–0.81)^c^
Did not purchase		0.33 (0.05–2.01)	3.97 (0.70–22.45)
Gas station		1 [Reference]	1 [Reference]
Perceived addictiveness of JUUL			
Moderately addictive	.02	1.80 (0.58–5.64)	1.95 (0.18–21.27)
Very addictive		1.33 (0.35–5.04)	7.05 (0.54–92.91)
Not at all addictive		1 [Reference]	1 [Reference]
Perceived harm of JUUL, compared with smoking			
Less harmful	<.001	4.08 (1.96–8.47)^e^	1.65 (0.53–5.16)
Just as much harm		4.30 (1.79–10.35)^d^	5.84 (1.71–19.92)^d^
Much more harm		11.21 (0.95–131.79)	28.20 (2.46–323.29)^d^
Much less harm		1 [Reference]	1 [Reference]
PSECDI dependence level^f^			
Low	.002	3.70 (1.41–9.72)^d^	3.10 (0.59–16.28)
Medium		4.96 (1.78–13.81)^d^	13.75 (2.48–76.26)^d^
High		4.47 (1.36–14.66)^c^	10.43 (1.66–65.42)^c^
Not dependent		1 [Reference]	1 [Reference]
Days of JUUL use in past 30 days	.001	0.96 (0.92–0.99)^c^	0.90 (0.85–0.96)^e^
Months of e-cigarette use	.006	0.81 (0.71–0.93)^d^	0.84 (0.70–1.00)
**Quit confidence^g^**			
PSECDI dependence level^f^			
Low	<.001	2.00 (0.63–6.33)	0.68 (0.23–2.02)
Medium		0.98 (0.33–2.90)	0.14 (0.04–0.41)^e^
High		0.50 (0.16–1.56)^c^	0.08 (0.02–0.26)^e^
Not dependent		1 [Reference]	1 [Reference]

Abbreviation: PSECDI, Penn State Electronic Cigarette Dependence Index.

a A 30-minute survey was developed by the study team and administered at 3 time points: July 2019, January 2020, and April 2020. Estimates of each variable level were determined by using binomial and multinomial logistic regression models.

b The question on quit importance was, “How important is it for you to quit electronic cigarette use now?” Akaike information criterion = 552.5; residual deviance = 480.5.

c *P* < .05; determined by χ^2^ test.

d *P* < .01; determined by χ^2^ test.

e *P* < .001; determined by χ^2^ test.

f Scores range from 0 to 20, where higher scores indicate greater dependence. Levels of dependence were categorized as not dependent (0–3), low dependence (4–8), medium dependence (9–12), and high dependence (≥13) (17).

g The question on quit confidence was, “How confident are you that you could quit electronic cigarette use now?” Akaike information criterion = 605.5; residual deviance = 589.5.

## Discussion

The current study surveyed adult JUUL users on their quit intentions, attempts, importance, and confidence and their perceptions of JUUL harm and addictiveness in 3 unique groups at 3 time points. A minority of our participants reported an intention to quit JUUL in the upcoming year or that they had ever attempted to quit JUUL use. Most of our sample perceived e-cigarettes to be less harmful than smoking but at least moderately addictive. Public health warnings about e-cigarette addictiveness have targeted JUUL because of its ability to deliver nicotine levels more similar to a combustible cigarette and at higher levels than most other e-cigarettes on the market (20). The actual addiction potential of e-cigarettes, however, remains under debate because levels of self-reported dependence on e-cigarettes are consistently lower among e-cigarettes users compared with cigarette smokers (17,21,22). In our sample, perceived addictiveness increased with level of dependence, suggesting that participants’ perceptions of JUUL addictiveness may be directly informed by awareness of their own addiction to e-cigarettes.

Higher perceived addictiveness was associated with more quit intentions, attempts, and importance, while higher perceived harm compared with smoking was associated only with more quit importance. Previous qualitative studies of adolescents and young adults found harm and addiction perceptions were primary reasons for future intentions to quit e-cigarette use and previous quit attempts (23,24). The link between addiction perceptions and quit intentions suggests that messaging about the addictiveness of e-cigarettes could increase public interest in quitting among adults, even for smokers using them as cigarette alternatives. This highlights the need for more evidence-based information about the addictiveness of e-cigarettes, including which e-cigarette product features or user characteristics increase risk for e-cigarette addiction.

Reported quit importance was highest in the third and final survey time point, which occurred in April 2020, after the e-cigarette or vaping use-associated lung injury (EVALI) outbreak that began in July 2019 and resolution of its cause (ie, black market tetrahydrocannabinol [THC] products contaminated with vitamin E acetate) in February 2020 (25). We did not measure exposure to EVALI information; however, we suspect that the increased media attention to the potential harms of e-cigarettes in late 2019 may have contributed to the increased quit importance in the survey data collected in January 2020. Although we did not find a significant increase in harm perceptions in our later cohorts, our assessment of relative harm compared with smoking may have missed JUUL users’ perceptions of general harm. Alternatively, the COVID-19 pandemic led to major shutdowns and a surge of health information in the US in March 2020, just before our final survey in April 2020. The increase in quit importance we observed may have been related to the attention to respiratory health and tobacco use during the pandemic and not e-cigarette messaging.

Higher e-cigarette dependence as measured on PSECDI was associated with more quit attempts, higher quit importance, and lower quit confidence, but not quit intentions. This finding, combined with the positive association between dependence and perceived addictiveness, suggests that JUUL users with higher levels of dependence are aware of their own difficulties with quitting and might be open to e-cigarette cessation interventions, even if they are not planning to quit on their own. Simply inquiring about interest in e-cigarette cessation or administering brief self-report dependence measures in clinical and community settings could be an easy and effective way to identify e-cigarette users who might be willing to make a quit attempt. However, our data show that quitting confidence is not high among JUUL users in general and is even lower among those with high levels of dependence. This points to the need for interventions that can boost quitting confidence and success.

Empirically supported treatments of e-cigarette cessation are lacking. A pilot trial showed promise for nicotine replacement therapy, tapering use, and self-guided methods for e-cigarette cessation among young adults (26). A randomized clinical trial among more than 2,000 young adult e-cigarette users found better cessation rates in a text-messaging program (24.1%) than in an assessment-only control condition after 7 months (18.6%) (27). The cessation program, This is Quitting, used a combination of social support and cognitive and behavioral coping skills training conducted entirely through text messages (27). Among our sample, those who attempted to quit primarily did it “cold turkey” and almost half of those who attempted reported that cessation lasted less than a month. Most previous research has focused on links between e-cigarette use and smoking cessation, rather than e-cigarette cessation (28). More research is needed to develop effective evidence-based cessation interventions, to increase awareness of e-cigarette cessation options among the public, and to identify those most in need of cessation programs.

The primary limitations of the current study are the small sample size and use of convenience sampling through MTurk. MTurk participants are not representative of the general US population, which limits our ability to make any population-based inferences from our results (29). However, MTurk is an ideal platform to recruit e-cigarette users because workers tend to be young and overrepresent substance users (30). The anonymity of MTurk facilitates honest and unbiased responses when stigmatized behaviors are being assessed, which cannot always be achieved in face-to-face laboratory studies. We also used numerous data validity and reliability checks. As with any survey study, we were not able to biochemically verify that participants were nicotine users. Purposely masking the true eligibility criteria for the study helped to identify respondents who were not current e-cigarette users. Participants had little incentive to lie about their use, given the many work opportunities on MTurk.

In conclusion, we found that e-cigarette quit intentions and attempts were low among our sample of US adult JUUL users and were associated with higher perceptions of JUUL addictiveness. Increased awareness of the potential addictiveness of e-cigarettes may drive quit intentions and attempts; however, more empirical data on e-cigarette addictiveness is needed to accurately inform the public. Public health priorities should include continuing to educate the public on the known risks of e-cigarettes, their potential role in assisting smoking cessation (31), developing methods for identifying and engaging those at risk for e-cigarette dependence, and developing effective and accessible methods for e-cigarette cessation.
